# Artificial Dim Light at Night during Pregnancy Can Affect Hormonal and Metabolic Rhythms in Rat Offspring

**DOI:** 10.3390/ijms232314544

**Published:** 2022-11-22

**Authors:** Zuzana Dzirbíková, Katarína Stebelová, Katarína Kováčová, Monika Okuliarová, Lucia Olexová, Michal Zeman

**Affiliations:** Department of Animal Physiology and Ethology, Faculty of Natural Sciences, Comenius University, Ilkovičova 6, 842 15 Bratislava, Slovakia

**Keywords:** ALAN, pregnancy, melatonin, corticosterone, vasopressin, thyroid hormones, metabolites, ontogeny, circadian rhythm, rat pups

## Abstract

Artificial light at night (ALAN) is considered an environmental risk factor that can interfere with the circadian control of the endocrine system and metabolism. We studied the impact of ALAN during pregnancy on the hormonal and biochemical parameters in rat pups at postnatal (P) days P3, P10, and P20. Control dams (CTRL) were kept in a standard light-dark regime, and ALAN dams were exposed to dim ALAN (<2 lx) during the whole pregnancy. A plasma melatonin rhythm was found in all CTRL groups, whereas in ALAN pups, melatonin was not rhythmic at P3, and its amplitude was lowered at P10; no differences were found between groups at P20. Plasma corticosterone was rhythmic at P20 in both groups, with decreased mesor in ALAN pups. Plasma thyroid hormones exhibited an inconsistent developmental pattern, and vasopressin levels were suppressed at the beginning of the dark phase at P20 in ALAN compared to CTRL. Glucose and cholesterol showed significant daily rhythms in CTRL but not in ALAN offspring at P3. Exposure to ALAN during pregnancy disturbed the development of daily rhythms in measured hormones and metabolites, suggesting that ALAN during pregnancy can act as an endocrine disruptor that can interfere with the normal development of the progeny.

## 1. Introduction

The endocrine system and metabolism are under strong circadian control that facilitates effective adaptive responses of the organism to regular changes in environmental conditions [1]. All important physiological systems display circadian oscillations that align physiological, metabolic, and behavioral rhythms together and with cycling environmental conditions. Such coordination of internal processes with predictable changes in the environment is an inevitable prerequisite for the efficient functioning of organisms.

The circadian system of mammals is hierarchically organized with the suprachiasmatic nucleus (SCN) of the hypothalamus as the master regulator at the top of the system [2]. The SCN is predominantly entrained by the light-dark cycle (LD) via intrinsically photosensitive ganglion cells that relay light information via the retinohypothalamic tract to the SCN [3]. Circadian rhythms in the body are synchronized by rhythmic outputs from the SCN mediated via the endocrine and autonomic nervous systems [2]. The photoperiod, or the LD cycle, used to be the most reliable environmental signal for the synchronization of circadian rhythms, but its reliability has recently been challenged by artificial light at night (ALAN) [4]. Light pollution has been increasing abruptly during the last decades as a result of the rapid growth of population and urbanization, as well as the introduction of new technologies, especially the use of energy-efficient light-emitting diodes [5].

Negative consequences of artificial or unexpected lighting, causing chronodisruption, have been examined in particular with relation to bright light during the evening [6], jet lag [7,8], or shift work [9,10]. However, recent studies suggest that a low intensity of ALAN lasting all night can interfere with vital physiological [11] and neural processes in humans [12]. Molecular mechanisms mediating the negative effects of ALAN are explored in animal experiments and include disruption of the circadian rhythms in metabolism [13,14], sleep [15], or the immune system [16,17]. The developmental aspects of chronodisruption have been less studied compared to mature animals and humans but can be important because maternal shift work can have negative consequences for offspring [18]. A meta-analysis documenting shift-working pregnant women showed a small risk of pregnancy outcomes such as low birth weight, preterm delivery, and small-for-gestational-age in relation to shift working [19]. The circadian system develops “in utero” through exposure to maternal cues [20,21]. The importance of maternal–fetal communication is well known for other environmental factors such as nutrition, stress, and hypoxia, resulting in the development of diseases in adulthood [22]. The risk of maternal exposure to constant light during the second part of pregnancy has recently been underlined by the possibility that suppressed maternal melatonin can modify the epigenome of the fetal liver [23,24].

The circadian system develops gradually during the first 2 weeks of postnatal life in rats [25,26] and during the first 2 months in humans [27,28]. The interaction between the circadian rhythms of the mother and offspring shapes the development of the SCN [29] and peripheral clocks [30]. Therefore, lighting conditions during pregnancy and the early postnatal period can influence physiological and behavioral rhythms in offspring [31]. Indeed, constant light or shifts of the LD regime can negatively affect the development of the circadian system [32], and the pharmacological doses of the hormone melatonin can synchronize the molecular clockwork in the fetal SCN but not in the liver [33]. The development of rhythms in vasopressin, as the output signal from the central clock, occurs before birth [34], suggesting its important role in the fetal circadian organization.

Our recent study in laboratory rats demonstrates that even dim ALAN (~2 lx), which can be commonly found in cities, disturbs the rhythmic expression of clock and clock-controlled genes in the SCN, eliminates the daily rhythms of plasma melatonin and testosterone, and vasopressin and disturbs the daily corticosterone rhythm [35]. Moreover, the daily rhythms of key metabolites and metabolic sensors were disturbed in mature male rats kept under identical lighting conditions [14]. It is unknown if such a level of dim ALAN during pregnancy can affect the development of daily rhythms in key hormonal and metabolic signals. Therefore, in this study, we analyzed daily rhythms in plasma concentrations of melatonin, corticosterone, thyroid hormones, vasopressin, and metabolites in offspring that were born to mothers exposed to dim ALAN during pregnancy and kept in a standard LD regime postnatally.

## 2. Results

**Figure 1:**
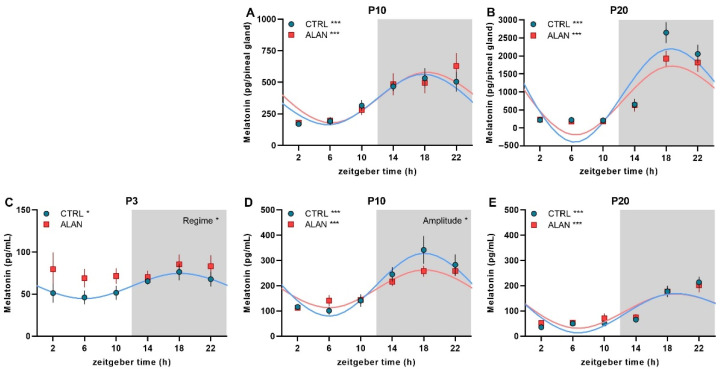
Effects of gestational dim ALAN (~2 lx) on the early development of daily melatonin rhythms. Pineal melatonin rhythms on postnatal day P10 (**A**) and P20 (**B**) and plasma melatonin rhythms on P3 (**C**), P10 (**D**), and P20 (**E**) in rat pups that were delivered by mothers exposed to either the control light–dark cycle (CTRL) or ALAN during pregnancy. Symbols represent the mean ± SEM, *n* = 3–6 rats per time point and group. The significant 24-h rhythms are fitted with a cosine curve for CTRL (blue solid line) and ALAN (red solid line) groups at *** *p* < 0.001 and * *p* < 0.05. Non-rhythmic data are not fitted with the cosine curve. Significant group differences in the rhythm amplitude and the main effects of the Regime from two-way ANOVA are indicated at * *p* < 0.05. Zeitgeber time 0 = light on. Grey area represents the dark phase. Note different scales in graphs.

On postnatal days P10 and P20, pineal melatonin (MEL) levels showed distinct daily rhythms in both control pups (CTRL; *p* < 0.001 for P10 and P20) and pups of dim light exposed mothers during pregnancy (ALAN; *p* < 0.001 for P10 and P20), with an acrophase in the middle of the dark phase (Figure 1A, B and Table 1). Plasma MEL levels were measured already on postnatal day P3, when a significant daily rhythm was found in the CTRL (*p* < 0.05) but not in the ALAN group (*p* = 0.51; Figure 1C, Table 1). Moreover, 3-day-old ALAN pups exhibited higher plasma MEL levels than controls (F_(1,58)_ = 6.68, *p* < 0.05; two-way ANOVA), and this difference was mainly found in the light phase (ALAN vs. CTRL: 73.3 ± 7.8 vs. 49.6 ± 5.2 pg/mL; *t* = 2.762, *p* < 0.05 with Bonferroni correction). On P10, distinct rhythms in plasma MEL were detected in both the CTRL (*p* < 0.001) and ALAN groups (*p* < 0.001), whereas the rhythm had a lower amplitude in ALAN compared to CTRL pups (*p* < 0.05; Figure 1D, Table 1). At the time of weaning (P20), daily rhythms in plasma MEL did not differ between CTRL and ALAN groups, and the overall patterns and concentrations were identical to those typical for adult rats (Figure 1E, Table 1).

**Figure 2:**
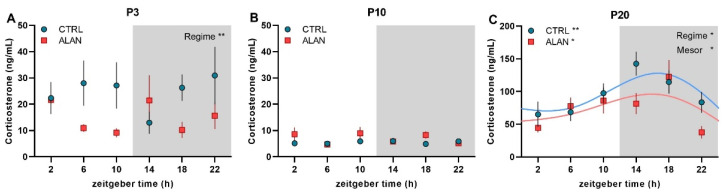
Effects of gestational dim ALAN (~2 lx) on the early development of daily corticosterone rhythms. The daily pattern of plasma corticosterone levels on postnatal day P3 (**A**), P10 (**B**), and P20 (**C**) in rat pups that were delivered by mothers exposed to either the control light–dark cycle (CTRL) or ALAN during pregnancy. Symbols represent the mean ± SEM, *n* = 5–6 rats per time point and group. The significant 24-h rhythms are fitted with a cosine curve for CTRL (blue solid line) and ALAN (red solid line) groups at ** *p* < 0.01 and * *p* < 0.05. Non-rhythmic data are not fitted with the cosine curve. A significant difference in mesor of the rhythms is indicated at * *p* < 0.05. Significant main effects of Regime from two-way ANOVA are indicated at ** *p* < 0.01, * *p* < 0.05. Zeitgeber time 0 = light on. Grey area represents the dark phase. Note different scales in graphs.

Plasma corticosterone concentrations, as a main output of the hypothalamus-pituitary-adrenal axis, did not exhibit significant daily rhythms in either CTRL or ALAN groups in P3 and P10 (Figure 2A,B and Table 1). However, two-way ANOVA revealed lower mean corticosterone levels in 3-day-old ALAN pups compared to controls (F_(1,58)_ = 9.25, *p* < 0.01; Figure 2A). On P20, daily corticosterone rhythms were detected in both the CTRL (*p* < 0.01) and ALAN groups (*p* < 0.05; Figure 2C), but mean corticosterone levels were lower in ALAN compared to CTRL pups, as indicated by differences in mesor (*p* < 0.05; Table 1) and confirmed by two-way ANOVA (factor regime: F_(1,59)_ = 4.36, *p* < 0.05; Figure 2C).

**Figure 3:**
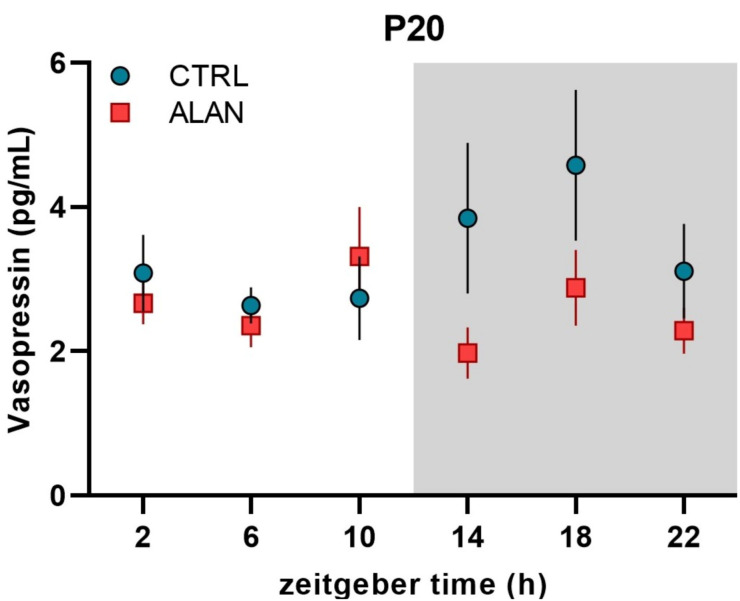
Effects of gestational dim ALAN (~2 lx) on the daily profile of plasma vasopressin levels in 20-day-old rat pups that were delivered by mothers exposed to either the control light–dark cycle (CTRL) or ALAN during pregnancy. Symbols represent the mean ± SEM, *n* = 5–6 rats per time point and group. Zeitgeber time 0 = light on. The grey area represents the dark phase.

Because of the low amount of available plasma, the daily pattern of plasma vasopressin was measured only on postnatal day P20. In both CTRL and ALAN groups, vasopressin levels did not exhibit daily rhythms (Figure 3 and Table 1), but two-way ANOVA revealed an interaction between the regime and phase of the LD cycle (F_(1,68)_ = 3.79, *p* = 0.056), demonstrating lower vasopressin levels in ALAN compared to CTRL pups specifically during the dark phase (ALAN vs. CTRL: 2.39 ± 0.24 vs. 3.85 ± 0.53 pg/mL; *p* < 0.01, Bonferroni posthoc comparison test).

**Figure 4:**
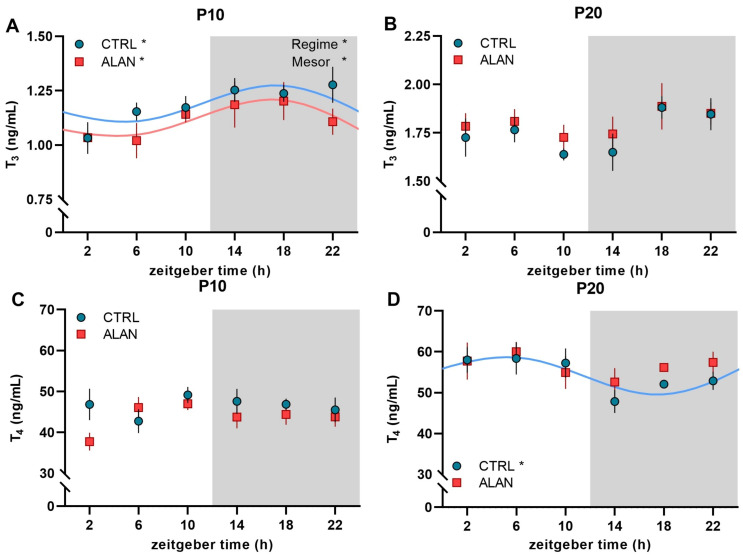
Effects of gestational dim ALAN (~2 lx) on the early development of daily rhythms in thyroid hormones. A daily profile of plasma triiodothyronine—T_3_ (**A**,**B**) and thyroxine—T_4_ (**C**,**D**) levels on postnatal days P10 (**A**,**C**) and P20 (**B**,**D**) in rat pups that were delivered by mothers exposed to either the control light–dark cycle (CTRL) or ALAN during pregnancy. Symbols represent the mean ± SEM, *n* = 4–6 rats per time point and group. The significant 24-h rhythms are fitted with a cosine curve for CTRL (blue solid line) and ALAN (red solid line) groups at * *p* < 0.05. Non-rhythmic data are not fitted with the cosine curve. Significant difference in mesor of the rhythms is indicated at * *p* < 0.05. Significant main effects of Regime from two-way ANOVA are indicated at * *p* < 0.05. Zeitgeber time 0 = light on. Grey area represents the dark phase. Note different scales in T_3_ (**A**,**B**).

Plasma thyroid hormones T_3_ and T_4_ exhibited an inconsistent developmental pattern. On P10, plasma T_3_ levels showed significant daily rhythms, with a peak during the dark time in both CTRL (*p* < 0.05) and ALAN groups (*p* < 0.05; Figure 4A and Table 2). However, the mean T_3_ levels were significantly lower in ALAN than in CTRL pups, as indicated by group differences in mesor (Table 2) and by two-way ANOVA (factor regime: F_(1,51)_ = 4.02, *p* = 0.05). In contrast to T_3_, the plasma T_4_ levels in P10 did not show a rhythmic profile, and no differences between CTRL and ALAN pups were found (Figure 4C). On P20 arrhythmic T_3_ concentrations were observed in both CTRL and ALAN groups (Figure 4B), whereas the T_4_ levels exhibited a significant daily rhythm in the CTRL (*p* < 0.05; Table 2), but not in the ALAN group (Figure 4D).

**Figure 5:**
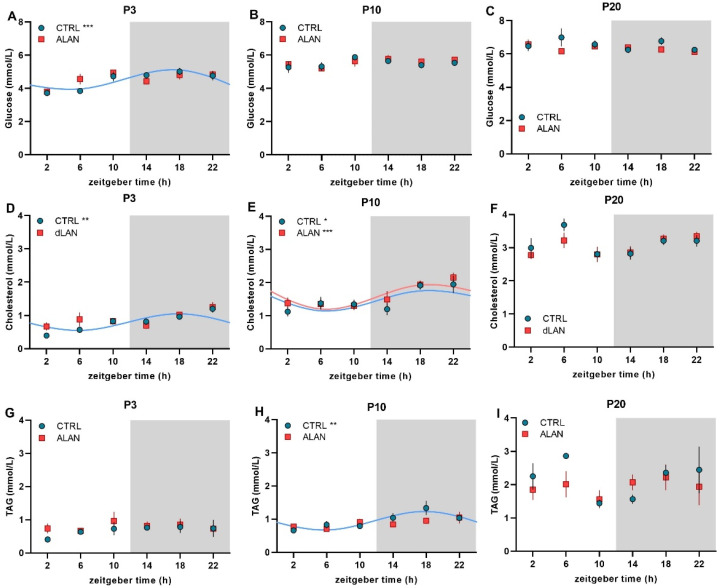
Effects of gestational dim ALAN (~2 lx) on the early development of daily rhythms in plasma metabolites. A daily profile of plasma glucose (**A**–**C**), cholesterol (**D**–**F**), and triacylglycerol (TAG) levels (**G**–**I**) on postnatal days P3 (**A**,**D**,**G**), P10 (**B**,**E**,**H**) and P20 (**C**,**F**,**I**) in rat pups that were delivered by mothers exposed to either the control light–dark cycle (CTRL) or ALAN during pregnancy. Symbols represent the mean ± SEM, *n* = 4–6 rats per time point and group. The significant 24-h rhythms are fitted with a cosine curve for CTRL (blue solid line) and ALAN (red solid line) groups at *** *p* < 0.001, ** *p* < 0.01, and * *p* < 0.05. Non-rhythmic data are not fitted with the cosine curve. Zeitgeber time 0 = light on. Grey areas represent the dark phase.

The 24-h profiles of plasma glucose, cholesterol, and triacylglycerol levels were analyzed in the CTRL and ALAN groups at three developmental stages. On P3, CTRL pups showed significant daily rhythms in glucose (*p* < 0.001; Figure 5A) and cholesterol (*p* < 0.01; Figure 5D), whereas both metabolites were arrhythmic in the ALAN group (Figure 5A,D, and Table 3). The triacylglycerol levels were arrhythmic in both groups at this developmental stage (Figure 5G and Table 3). On P10, the rhythmicity in glucose levels was lost in the CTRL group (Figure 5B), but CTRL pups showed daily rhythms in cholesterol (*p* < 0.05; Figure 5E) and triacylglycerol levels (*p* < 0.01; Figure 5H and Table 3). In the ALAN group, only cholesterol levels displayed a significant rhythm at this developmental stage (*p* < 0.01; Figure 5E). On P20, at the time of weaning, none of the measured metabolites exhibited daily oscillations in either the CTRL or ALAN groups (Figure 5C,F,I and Table 3).

## 3. Discussion

The plasma melatonin rhythm was evident for all tested ages in CTRL pups, whereas in the ALAN pups, plasma melatonin was not rhythmic at P3, and its amplitude was lowered at P10 compared to CTRL. No differences were found between the CTRL and ALAN groups at P20. No effect of prenatal ALAN was found on melatonin synthesis in the pineal gland.

The differences in plasma melatonin concentrations in P3 pups between both groups probably reflect the suppression of melatonin in pregnant ALAN mothers because, at this age, the pineal gland does not synthesize melatonin rhythmically [36]. Although we did not measure the melatonin levels in pregnant female rats in this study to avoid an additional stress stimulus, our previous studies convincingly showed that the night-time illuminance applied in this experiment suppressed plasma melatonin rhythm in adult male rats [35,37]. In previous studies, higher levels of ALAN were applied to explore the effect of ALAN, from 5 lx [13,15] to 20 lx [38]. Autonomous rhythmic melatonin biosynthesis starts in rats at postnatal day 5 in the pineal gland [36], and therefore, the melatonin plasma levels in 3-day-old offspring reflect maternal melatonin. This is supported by the fact that melatonin is present in milk, and its levels exhibit a distinct rhythm in humans [39] and rat milk [36]. The absence of a melatonin rhythm in the plasma of ALAN pups at P3 is associated with higher levels during the daytime and probably reflects a less synchronized circadian system in the ALAN mothers. This finding, together with the lower amplitude of the plasma melatonin rhythm at P10 in ALAN compared to CTRL offspring, suggests a long-term effect of ALAN during pregnancy on the plasma melatonin rhythms in offspring. Taken together, our results show that ALAN during pregnancy significantly affected the development of melatonin rhythmicity in offspring, although, after delivery, the mothers and their progeny were kept under standard LD conditions. Further studies should therefore address whether postnatal ALAN can interfere with the observed changes or even augment them.

As far as we know, this is the first study demonstrating that exposure of pregnant mothers to ALAN can affect the development of plasma hormone rhythmicity in offspring kept postnatally, together with their mothers, on a regular LD cycle. Another model of maternal chronodisruption, chronic shifts of photoperiod during pregnancy, altered the circadian organization of pregnant mothers, but their melatonin rhythm was preserved, although with a lower amplitude. Surprisingly, in adult male offspring exposed to shifts prenatally, the melatonin rhythm was eliminated, whereas it was present in control animals [40]. The design of our study did not allow us to evaluate intersex differences in plasma melatonin levels in response to prenatal exposure to ALAN, and this aspect, together with the long-term effects of prenatal ALAN on physiology and behavior, is an object of our follow-up experiments.

Maternal melatonin serves as a hormonal signal from the mother to the fetus [21,33], and therefore, the suppressed plasma melatonin rhythm at P3 in ALAN offspring can influence the development of the central oscillator and other rhythms in physiology and behavior. The importance of the melatonin rhythm during pregnancy is illustrated by the fact that its levels in the circulation of pregnant women increase substantially from week 32 of pregnancy and are normalized only after delivery [41]. Similarly, in rats, night-time melatonin levels increase during the third week of pregnancy, peak immediately before parturition, and return to non-pregnant levels by postpartum day 2 [42]. Data on the melatonin profile in pregnant females kept under disturbed lighting conditions are rather limited, although they can be important because of their long-term consequences on their progeny. Maternal melatonin can influence the fetus in different ways because it passes through the placenta into the fetal circulation [43], and melatonin receptors were found in the different organs of the fetus, including the SCN [44]. Administration of melatonin to pregnant female rats with suppressed endogenous melatonin production entrains clock gene expression profile in the SCN of offspring. Interestingly, the same treatment failed to affect clock gene expression in the liver [33], suggesting that the central but not hepatic oscillators in the foetus are sensitive to this hormone.

Corticosterone is another hormonal signal involved in maternal–fetal communication [45]. In our experiment, the daily rhythm in corticosterone appeared at P20, with decreased mesor in ALAN compared to CTRL offspring. In line with published data [30], we found a distinct developmental pattern in plasma corticosterone levels, with high levels after birth, decreased concentrations at P10, and an adult-like pattern at P20. This developmental pattern can be related to the important role of this hormone in the perinatal development and maturation of several organs, such as the lungs. Indeed, the development of circadian rhythms is delayed in the lungs compared to other organs and was found at postnatal day 10 in Per1-luc mice [46]. From this point of view, lower corticosterone levels in ALAN compared to CTRL at P3 can have negative consequences, especially on organs that continue in their development after delivery, such as the brain and the kidney. A shift of the photoperiod during gestation in mothers affected the corticosterone levels and rhythmicity in young adult male offspring, simultaneously modifying several parameters of kidney function, such as creatinine, urea, and blood urea nitrogen levels [40].

In our study, we measured plasma vasopressin concentrations only at the time of weaning because of the low volume of plasma in earlier developmental stages. We found lower levels of circulating vasopressin in ALAN compared to CTRL during the dark phase at P20. As 20-day-old rats already also consume solid food and drink water [47], the lower vasopressin levels may be associated with changes in the control of the water balance. In mature male rats, we found a daily rhythm of circulating vasopressin in control but not ALAN-exposed rats, with an acrophase during the dark time [35]. In mature animals, vasopressin rhythmicity is related to anticipatory thirst, which is regulated by circadian clocks [48] and is disrupted after ALAN exposure [35]. So far, no data are available for pups, although it is known that cord blood vasopressin concentrations are elevated in neonates [49], and the highest levels of vasopressin were measured in fetuses and new-borns after hypoxia [50]. The physiological significance of vasopressin during development is currently poorly understood, but the presence of vasopressin receptors in different brain areas, vasculature, and kidney [51] suggest its important role during early ontogeny, as demonstrated in mature animals and humans.

The plasma thyroid hormones T_3_ and T_4_ exhibited an inconsistent development of rhythmic patterns in our study. Maternal thyroid hormones cross the placenta to the fetus and play a key role during the first half of fetal development in humans and laboratory rodents [52]. The fetal thyroid gland starts secreting thyroid hormones from mid-gestation in humans and from embryonic day 17 in rats [53,54]. The maturation of the hypothalamic-pituitary control of the thyroid gland is completed at birth in humans but is delayed until approximately postnatal day P12-15 in altricial rats and mice [55,56]. Therefore, the daily rhythm found at P3 in both groups probably reflects the maternal contribution. Lower levels in ALAN compared to CTRL rats may have consequences on the maturation of metabolic processes, which are markedly affected by thyroid hormones.

The circulating concentration of thyroid stimulating hormone (TSH) showed a clear circadian rhythm, with higher levels during the inactive phase [57,58], but only a low amplitude or no rhythm of T_3_ and T_4_ have been reported in rat serum [59]. The SCN may modulate thyroid hormone secretion by affecting the neuroendocrine control of TSH release through thyrotropin-releasing hormone neurons in the paraventricular nuclei and via autonomic input into the thyroid gland [60]. Moreover, the target tissue can modulate daily rhythms of thyroid hormones via activating local metabolism. Deiodinase 2, which converts T_4_ into the biologically much more active T_3_, has been shown to exhibit rhythmic activity in several tissues, including the pituitary and hypothalamus [61]. The appearance of the daily rhythm in T_4_ in CTRL on P20 can be important because of the high potential of thyroid hormones to synchronize peripheral circadian clocks and metabolism in different organs since almost all organs contain receptors for thyroid hormones [62].

In our study, the plasma metabolites exhibited the expected developmental pattern. Glucose and cholesterol showed significant daily rhythms in CTRL but not in ALAN offspring. This difference between groups is most likely determined predominantly by mothers. In our recent study with mature male rats [14], the same intensity of ALAN eliminated daily oscillations of plasma metabolites in the ALAN but not in the CTRL group. To our knowledge, no studies dealing with the rhythmicity of hormones and metabolites in pregnant females exposed to ALAN are available, requiring further research. A limitation of our study is that we were not able to evaluate intersex differences in response to prenatal exposure to ALAN. Thus, it is possible that one sex is more sensitive to prenatal ALAN exposure than the other, and this aspect should be investigated in future studies.

In conclusion, the daily rhythms in measured hormones and metabolites developed gradually during the early postnatal period. The lower levels of corticosterone at P3 and T_3_ at P10 in the ALAN compared to the CTRL group can have negative developmental consequences, especially on organs that continue in their development after delivery, such as the brain and the kidney. The delayed daily rhythm in plasma melatonin, as the direct output of the central clocks, may reflect the delayed development of the circadian system in response to ALAN. Together, exposure to ALAN during pregnancy disturbed the development of measured hormones and metabolites, suggesting that ALAN during pregnancy can act as an endocrine disruptor that can interfere with the normal development of the progeny.

## 4. Materials and Methods

### 4.1. Animals

We used mature Wistar rats, 28 females and 8 males, as the parental generation. The weight of the animals at the beginning of the experiment was 203 ± 2 g and 311 ± 4 g (mean ± SEM) for females and males, respectively. Rats were housed in plastic cages (four animals per cage) under standard lighting regime LD 12:12. During the entire experiment, the standard pellet diet and water were available ad libitum. The temperature in the experimental rooms was maintained at 22 ± 1 °C, with a relative humidity of 55–65%.

Animals were obtained from the breeding station of the Institute of Experimental Pharmacology and Toxicology, Centre of Experimental Medicine, Slovak Academy of Sciences, Dobra Voda, Slovakia. The experiment was approved by the Ethical Committee for the Care and Use of Laboratory Animals at Comenius University in Bratislava, Slovak Republic, and by the State Veterinary Authority (Ro-3906/19-221/3) in the Slovak Republic.

### 4.2. Experimental Design

After the acclimatization period (14 days), vaginal smears were taken from females to identify the estrous cycle phase [63]. In proestrus, the female was housed with the male overnight. The presence of spermatozoa in the vaginal smear on the next morning was designated as pregnancy day 0, and on that day, females were randomly divided into two groups—a control group (CTRL; *n* = 9) and a group exposed to ALAN (*n* = 11) (Table 4). Females without spermatozoa in the vaginal smears (*n* = 8) were excluded from the experiment.

During pregnancy, the CTRL dams were housed under the standard LD 12:12 regime, with lights on at 8:00 a.m. and total darkness during the night. The ALAN dams were exposed to LD 12:12, with lights on at 8:00 a.m., but during the dark phase, females were exposed to dim artificial light (<2 lx). The light on in both groups was designed as Zeitgeber time (ZT) 0. During the light phase in both groups, broad-spectrum ceiling lighting with an intensity of ~300 lx and a color temperature of 2900 K was used. Lighting conditions were measured with a spectrophotometer CL-500A (Konica Minolta Sensing Europe BV, Bremen, Germany) at the level of the animal’s cage. Two days before the expected delivery, each female was housed separately in a plastic cage. After delivery, the dams with their pups from the ALAN group were relocated into an LD regime with darkness during the night, together with CTRL dams.

The day of birth was designated as postnatal day 0 (P0). All descriptive parameters for dams and pups are listed in Table 4.

Pups were sacrificed on postnatal day 3 (P3, CTRL *n* = 36, ALAN *n* = 35), postnatal day 10 (P10, CTRL *n* = 36, ALAN *n* = 36), and postnatal day 20 (P20, CTRL *n* = 36, ALAN *n* = 36) during the 24-h cycle in 4-h intervals at six time points (ZT2, 6, 10, 14, 18, and 22). For each time point, six pups from different dams were included in P3 and P10. At P20 in the ALAN group, six pups from five mothers were used at each time point. At P20 in the CTRL group, we used six pups from five dams only during dark phase sampling (ZT14, 18, 22); during the light phase, six pups from six dams at each time point were included. The pups were taken randomly from the home cage. Sampling during the dark phase was performed under a red light. Blood was collected into heparinized tubes, centrifuged at 2500× *g* for 10 min at 4 °C, and stored at −20 °C until analysis. Pineal glands were extirpated only from pups at P10 and P20 and stored at −80 °C until melatonin extraction.

### 4.3. Melatonin Assay

Melatonin (MEL) from the pineal gland and the plasma was measured by a radioimmunoassay (RIA). The MEL from the pineal gland was extracted with methanol; dried extracts were reconstituted in tricine buffer (pH 5.5) and measured by RIA as described previously [64]. The MEL antibody (G/S/704-6483, Stockgrand Ltd., University of Surrey, Guilford, UK) and (O-methyl-3H)-labeled MEL tracer (NET801250UC, PerkinElmer, Boston, MA, USA, specific activity 2790 GBq/mmol (75.4 Ci/mmol)) were used. Plasma MEL was measured within three assays, and pineal extract samples were measured in two assays. The intra-assay variation coefficient was lower than 8.5%, and the inter-assay variation coefficient was below 12.1%. The sensitivity of the assay was 0.5 pg of MEL per tube.

### 4.4. Corticosterone and Thyroid Hormones Assays

Plasma corticosterone levels were measured using a Corticosterone rat/mouse RIA kit (RIA-1364, DRG Instruments GmBH, Marburg, Germany). All samples were measured in three assays. The control samples with low and high concentrations were within the manufacturer´s range; the intra-assay variation coefficients were lower than 7%, and the inter-assay variation coefficient was below 10%.

Plasma thyroid hormones were measured by RIA kits for triiodothyronine (T_3_) and thyroxine (T_4_) (RK-609CT, RK-11CT1, Izotop, Budapest, Hungary), according to the manufacturer´s instructions. Only plasma samples from the P10 and P20 were measured for T_3_ and T_4_ because of the low amounts of plasma in P3 pups. The levels of T_3_ hormone were measured in one assay. The control samples were within the manufacturer´s range, and the intra-assay variation coefficient was below 3%. The T_4_ hormone levels were also measured in one assay; the control sample was in the expected range, and the intra-assay variation coefficient was below 2%.

### 4.5. Vasopressin Assay

The plasma vasopressin level was measured using the Rat ADH (antidiuretic hormone) ELISA kit (ER0712, Wuhan Fine Biotech Co., Ltd., Wuhan, China) according to the manufacturer´s instructions. We measured vasopressin in plasma at P20 because of the low amounts of plasma in P3 and P10 pups. The intra-assay coefficient was below 3%.

### 4.6. Plasma Glucose, Cholesterol, and Triacylglycerols Measurement

In the plasma samples, glucose (GLU), cholesterol (CHOL), and triacylglycerols (TAG) were determined enzymatically with commercial kits (ErbaLachema, BIO-LA-TEST, Brno, Czech Republic), according to the manufacturer´s instructions. The method was modified for 96-well plates for all metabolites.

### 4.7. Statistical Analysis

Data were analyzed in GraphPad Prism 9 (San Diego, CA, USA) and the R software (R Core Team, 2020). A cosine curve was fitted to the data by a linear least-squares regression method, and a significant 24-h rhythm was detected with an F-test by the rejection of the zero-amplitude hypothesis using an online cosinor analysis [65]. Mesor (the 24-h time series mean), amplitude (one-half the peak-to-trough difference), and acrophase (the peak time of the fitted curve) of the significant rhythms, and their 95% confidence intervals were derived from the cosinor analysis using the R packages “cosinor” and “cosinor2”. The mesor, acrophase, and amplitude of significantly rhythmic variables were compared between CTRL and ALAN groups on the basis of 95% confidence intervals and by Wald tests. To evaluate group differences in non-rhythmic variables, two-way analysis of variance (ANOVA) was used with the main factors: regime (comparison of CTRL and ALAN), ZT, or a phase of the LD cycle, as well as the interaction of these factors. For data that did not fit the normal distribution, logarithmic transformation was applied. Data are expressed as the mean ± standard error of the mean (SEM).

## Figures and Tables

**Table 1:** Cosinor analysis of daily rhythms for plasma melatonin (MEL), corticosterone (CORT), and vasopressin (VP) levels in rat pups that were delivered by mothers exposed to either the control light–dark cycle (CTRL) or dim ALAN during pregnancy. Rats were sampled at three developmental stages, on postnatal days P3, P10, and P20.

	Mesor	Amplitude	Acrophase hh:mm (ZT)	R^2^	*F*-Test	*p*-Value
P10 Pineal MEL (pg/gland)						
CTRL	358.6 ± 48.6	203.1 ± 134.9	17:30 ± 1:17	0.55	*F*_(2,28)_ = 17.27	**0.000**
ALAN	383.5 ± 62.9	203.5 ± 116.7	18:28 ± 1:42	0.41	*F*_(2,30)_ = 10.57	**0.000**
P20 Pineal MEL (pg/gland)						
CTRL	972.4 ± 207.8	1349.6 ± 1047.2	19:05 ± 0:48	0.73	F_(2,29)_ = 38.43	**0.000**
ALAN	825.9 ± 164.7	1010.9 ± 778.3	19:19 ± 0:52	0.71	*F*_(2,30)_ = 36.28	**0.000**
P3 Plasma MEL (pg/mL)						
CTRL	59.7 ± 6.9	15.2 ± 5.5	18:08 ± 2:27	0.23	*F*_(2,32)_ = 4.78	**0.015**
ALAN	-	-	-	0.04	*F*_(2,32)_ = 0.69	0.507
P10 Plasma MEL (pg/mL)						
CTRL	204.3 ± 26.1	125.5 ± 88.6	18:06 ± 1:07	0.57	*F*_(2,33)_ = 22.21	**0.000**
ALAN	187.9 ± 16.1	75.5 ± 52.7 *	18:09 ± 1:09	0.56	*F*_(2,33)_ = 21.05	**0.000**
P20 Plasma MEL (pg/mL)						
CTRL	97.8 ± 17.0	85.6 ± 61.4	19:46 ± 1:03	0.60	*F*_(2,32)_ = 24.04	**0.000**
ALAN	104.8 ± 17.2	74.1 ± 49.8	19:41 ± 1:15	0.52	*F*_(2,33)_ = 17.78	**0.000**
P3 Plasma CORT (ng/mL)						
CTRL	-	-	-	0.02	*F*_(2,32)_ = 0.32	0.728
ALAN	-	-	-	0.03	*F*_(2,32)_ = 0.42	0.664
P10 Plasma CORT (ng/mL)						
CTRL	-	-	-	0.01	*F*_(2,33)_ = 0.23	0.792
ALAN	-	-	-	0.00	*F*_(2,33)_ = 0.05	0.949
P20 Plasma CORT (ng/mL)						
CTRL	95.3 ± 13.1	37.0 ± 18.5	14:57 ± 1:54	0.32	*F*_(2,33)_ = 7.69	**0.002**
ALAN	74.6 ± 14.5 *	28.5 ± 8.1	13:58 ± 2:46	0.19	*F*_(2,32)_ = 3.77	**0.034**
P20 Plasma VP (pg/mL)						
CTRL	-	-	-	0.11	*F*_(2,33)_ = 2.05	0.145
ALAN	-	-	-	0.01	*F*_(2,33)_ = 0.16	0.852

Estimates of mesor (the time series mean), amplitude (one-half the peak-to-trough difference), and acrophase (the peak time of the fitted curve) ± 95% confidence limits, ZT—zeitgeber time. R2, F, and *p*-values describe the significance of 24-h rhythms. Differences in mesor, amplitude, and acrophase between the CTRL and ALAN groups were evaluated based on 95% confidence intervals and using Wald tests. * *p* < 0.05.

**Table 2:** Cosinor analysis of daily rhythms for plasma triiodothyronine (T_3_) and thyroxine (T_4_) levels in rat pups that were delivered by mothers exposed to either the control light–dark cycle (CTRL) or dim ALAN during pregnancy. Rats were sampled at three developmental stages, on the postnatal days P3, P10, and P20.

	Mesor	Amplitude	Acrophase hh:mm (ZT)	R^2^	*F*-Test	*p*-Value
P10 Plasma T_3_ (ng/mL)						
CTRL	1.18 ± 0.05	0.14 ± 0.03	16:22 ± 2:58	0.19	*F*_(2,30)_ = 3.43	**0.045**
ALAN	1.12 ± 0.05 *	0.15 ± 0.04	15:42 ± 2:49	0.21	*F*_(2,27)_ = 3.55	**0.045**
P20 Plasma T_3_ (ng/mL)						
CTRL	-	-	-	0.15	*F*_(2,32)_ = 2.8	0.076
ALAN	-	-	-	0.06	*F*_(2,32)_ = 0.96	0.393
P10 Plasma T_4_ (ng/mL)						
CTRL	-	-	-	0.03	*F*_(2,32)_ = 0.47	0.627
ALAN	-	-	-	0.13	*F*_(2,30)_ = 2.16	0.133
P20 Plasma T_4_ (ng/mL)						
CTRL	54.4 ± 2.3	6.2 ± 2.05	4:39 ± 2:29	0.21	*F*_(2,32)_ = 4.28	**0.022**
ALAN	-	-	-	0.07	*F*_(2,32)_ = 1.22	0.310

Estimates of mesor (the time series mean), amplitude (one-half the peak-to-trough difference), and acrophase (the peak time of the fitted curve) ± 95% confidence limits, ZT-zeitgeber time. R2, F, and *p*-values describe the significance of 24-h rhythms. Differences in mesor, amplitude, and acrophase between the CTRL and ALAN groups were evaluated based on 95% confidence intervals and using Wald tests. * *p* < 0.05.

**Table 3:** Cosinor analysis of daily rhythms for plasma glucose (GLU), cholesterol (CHOL), and triacylglycerol (TAG) levels in rat pups that were delivered by mothers exposed to either the control light–dark cycle (CTRL) or dim ALAN during pregnancy. Rats were sampled at three developmental stages, on postnatal days P3, P10, and P20.

	Mesor	Amplitude	Acrophase hh:mm (ZT)	R^2^	*F*-Test	*p*-Value
P3 Plasma GLU (mmol/L)						
CTRL	4.48 ± 0.18	0.66 ± 0.41	16:02 ± 1:28	0.46	*F*_(2,31)_ = 13.29	**0.000**
ALAN	-	-	-	0.13	*F*_(2,29)_ = 2.1	0.141
P10 Plasma GLU (mmol/L)						
CTRL	-	-	-	0.08	*F*_(2,32)_ = 1.48	0.242
ALAN	-	-	-	0.09	*F*_(2,29)_ = 1.52	0.236
P20 Plasma GLU (mmol/L)						
CTRL	-	-	-	0.03	*F*_(2,33)_ = 0.43	0.656
ALAN	-	-	-	0.01	*F*_(2,33)_ = 0.21	0.814
P3 Plasma CHOL (mmol/L)						
CTRL	0.8 ± 0.09	0.25 ± 0.13	17:40 ± 1:57	0.34	*F*_(2,29)_ = 7.54	**0.002**
ALAN	-	-	-	0.08	*F*_(2,29)_ = 1.26	0.300
P10 Plasma CHOL (mmol/L)						
CTRL	1.48 ± 0.16	0.34 ± 0.11	19:47 ± 2:30	0.21	*F*_(2,32)_ = 4.33	**0.022**
ALAN	1.60 ± 0.13	0.41 ± 0.23	19:50 ± 1:45	0.40	*F*_(2,29)_ = 9.72	**0.001**
P20 Plasma CHOL (mmol/L)						
CTRL	-	-	-	0.08	*F*_(2,33)_ = 1.41	0.258
ALAN	-	-	-	0.08	*F*_(2,33)_ = 1.4	0.261
P3 Plasma TAG (mmol/L)						
CTRL	-	-	-	0.10	*F*_(2,29)_ = 1.57	0.226
ALAN	-	-	-	0.04	*F*_(2,28)_ = 0.54	0.590
P10 Plasma TAG (mmol/L)						
CTRL	0.94 ± 0.1	0.29 ± 0.14	17:27 ± 1:54	0.31	*F*_(2,32)_ = 7.33	**0.002**
ALAN	-	-	-	0.11	*F*_(2,29)_ = 1.8	0.183
P20 Plasma TAG (mmol/L)						
CTRL	-	-	-	0.14	*F*_(2,33)_ = 2.78	0.077
ALAN	-	-	-	0.02	*F*_(2,33)_ = 0.35	0.710

Estimates of mesor (the time series mean), amplitude (one-half the peak-to-trough difference), and acrophase (the peak time of the fitted curve) ± 95% confidence limits, ZT—zeitgeber time. R2, F, and *p*-values describe the significance of 24-h rhythms.

**Table 4:** Parameters of pregnant dams and characteristics of their pups until postnatal day 1. Dams were maintained either under a control light-dark cycle (CTRL) or at dim light during the night (ALAN).

	CTRL	ALAN	*p*-Value
Pregnant females (n)	9	11	
Weight of dams at G0 (mean ± SEM)	220.89 ± 3.83 g	228.82 ± 4.73 g	0.223
Weight of dams at G20 (mean ± SEM)	354.44 ± 7.31 g	365.64 ± 11.63 g	0.450
Weight gain between G0 and G20 (mean ± SEM)	60.63 ± 3.07%	59.57 ± 2.86%	0.802
Total number of pups (n)	108	132	
Litter size/dam (mean ± SEM)	12 ± 1.12	12 ± 0.77	0.849
Weight of pup at P1 (mean ± SEM)	6.59 ± 0.27 g	6.71 ± 0.22 g	0.331
Number of pups in P3 (Male/Female) (n)	18/18	22/13	
Number of pups in P10 (Male/Female) (n)	14/22	23/13	
Number of pups in P20 (Male/Female) (n)	20/16	17/19	

G—gestational day, P—postnatal day.

## Data Availability

Not applicable.
